# The complete chloroplast genome of diploid *Hieracium tomentosum* (Asteraceae, Cichorieae)

**DOI:** 10.1080/23802359.2026.2728749

**Published:** 2026-10-22

**Authors:** Daniela Calzoni, Giacomo Baldesi, Fabio Palumbo, Giulia Ginelli, Simone Orsenigo

**Affiliations:** ^a^ Department of Earth and Environmental Sciences, University of Pavia, Pavia, Italy; ^b^ Department of Agronomy Food Natural resources Animals and Environment (DAFNAE), University of Padova, Legnaro, Italy

**Keywords:** *Hieracium tomentosum*, Hieraciinae, Cichorieae, Asteraceae, apomixis

## Abstract

*Hieracium* is a megadiverse genus within the family Asteraceae, in the tribe Cichorieae subtribe Hieraciinae. We report here the complete chloroplast genome of the diploid *Hieracium tomentosum* L. The assembled plastome measures 153,218 bp in total length with one large single-copy (LSC, 84,604 bp), one small single-copy (SSC, 18,438 bp), and two inverted repeat (IR, 25,088 bp) regions. It comprehends 127 genes, including 83 protein-coding genes, 8 rRNA genes, and 36 tRNA genes. Phylogenomic inference of 31 species within Cichorieae supports its placement within apomictic polyploid genus *Hieracium*, it highlights monophyly of Hieraciinae and its evolutionary relatedness to the subtribe Cichorieae. These data provide an important baseline for the systematics and further research of Asteraceae.

## Introduction

*Hieracium* is a megadiverse genus within the family Asteraceae (tribe Cichorieae), and its evolutionary history has been challenging to tackle due to processes such as hybridization, polyploidization, and apomixis which obscure species boundaries. It consists of only around 30 known diploids (2*n* = 2*x* = 18; Baldesi et al. 2025) and thousands of apomictic polyploid taxa. The *Hieracium tomentosum* series (Van Es and Tison 2018) (sect. *Andryaloidea* Monnier) is distinguished by the strongly plumose hairs, forming a dense felty whitish indumentum and includes a few species distributed from the Western Alps to the Central Apennines (Figure 1). Within the series, presently under investigation, at least one diploid species, *Hieracium tomentosum* L., has been identified, together with several triploid apomictic species. Diploid *H. tomentosum* has a narrow distribution restricted to the French–Italian border in the Maritime and Ligurian Alps and typically grows in rocky calcareous habitats.

**Figure 1.**
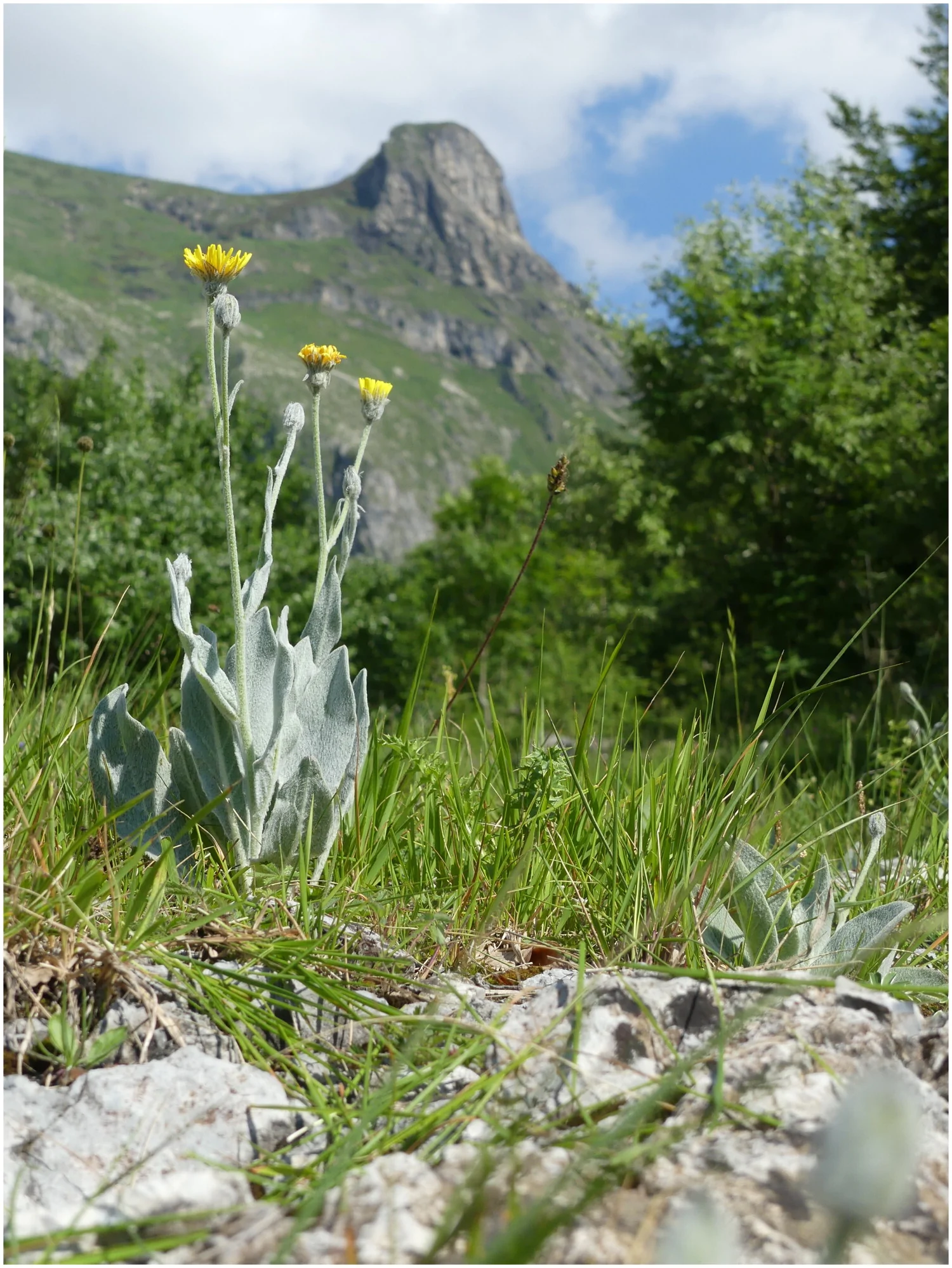
Photograph of diploid *Hieracium tomentosum* L. taken by Simone Orsenigo.

Only a few phylogenetic studies on *Hieracium* based on nuclear and plastidial markers have been conducted (Krak et al. 2013; Chrtek et al. 2020; Fehrer et al. 2021) and these have not yet fully resolved the complex evolutionary patterns within the genus. Despite its considerable diversity and taxonomical significance, only 10 annotated plastome sequences have been deposited in online databases, but are yet unpublished in formal research (Li et al. 2023, unpublished). On the contrary, more than 150 plastomes of tribe Cichorieae have been fully sequenced, representing eight subtribes (Cichoriinae, Crepidinae, Lactucinae, Hieraciinae, Hyoseridinae, Hypochaeridinae, Scorzonerinae, Scolyminae). Here, we present the complete chloroplast genome of diploid *H. tomentosum* and assess its phylogenomic positioning among Hieraciinae and Cichorieae, which represents an important step for phylogenomic and comparative studies of this megadiverse polyploid genus, as well as for broader evolutionary research.

## Materials and methods

Fresh leaves of *H. tomentosum* were collected in Prato Nevoso (44.2167 N; 7.7733 E) (Figure 1) and stored at −20 °C until DNA extraction. Voucher specimen is stored at the *Herbarium Universitatis Ticinensis* of the University of Pavia (PAV, contact person: Simone Orsenigo, email: simone.orsenigo@unipv.it) under voucher code PAV-H201826. Genomic DNA was extracted using the DNeasy Plant Mini Kit (Qiagen, Hilden, Germany). Quantity and quality were assessed using a Qubit 4 fluorometer with the dsDNA BR Assay Kit (Thermo Fisher Scientific, Waltham, MA) and with Genomic DNA ScreenTape reagents on the 4150 TapeStation system (Agilent Technologies, Santa Clara, CA), respectively. Libraries were prepared with the Illumina DNA Prep kit (Illumina Inc., San Diego, CA) and sequenced (2 × 150 bp) on an Illumina NovaSeq X Plus platform (Illumina, San Diego, CA).

Raw reads quality was assessed with FastQCv0.12.1 (Andrews 2010). The chloroplast genome was assembled using GetOrganelle v1.7.7.1 (Jin et al. 2020). Sequence length, coverage, and structure were visually inspected with Bandage (Wick et al. 2015). Genome annotation was carried out using the GeSeq web interface with default parameters for CDS and rRNA prediction (Tillich et al. 2017), supported by the third-party annotator Chloe v.0.1.0 for tRNA. The circular chloroplast genome map was obtained with the help of CPGView, as well as splicing genes’ structures (Liu et al. 2023). Finally, the raw reads and metadata of *H. tomentosum* were submitted to NCBI (SRR36998144 and SAMN54689699) under BioProject PRJNA1373174, along with GenBank accession numbers for annotated sequence PX775863.

To assess its phylogenomic relationships with related species, we selected 30 species from the tribe Cichorieae on the NCBI database. Whole plastome sequences were aligned using MAFFT (Katoh et al. 2002) with default parameters, and ambiguously aligned regions were removed using trimAl v1.5 with -nogaps option (Capella-Gutiérrez et al. 2009). Finally, we performed a maximum-likelihood (ML) analysis using IQ-TREE2 with model TVM+F+I+G4 with 1000 bootstrap replicates and SH-aLRT support (-B 1000 -alrt 1000) (Minh et al. 2020), setting *Tragopogon dubius* Scop. and *Scorzonera humilis* L. (*Scorzonerinae*) as outgroups.

## Results

The plastome of *H. tomentosum* exhibits the typical quadripartite structure characteristic of chloroplast genomes (Figure 2). The assembled circular genome is 153,218 bp in length with a GC content of 38%, and shows an average read mapping depth of 738× (Supplementary Figure 1). It consists of a large single-copy (LSC) region of 84,604 bp and a small single-copy (SSC) region of 18,438 bp. These are separated by two inverted repeat (IR) regions, each 25,088 bp long. Genome annotation identified a total of 127 genes, including 83 protein-coding genes, 8 rRNA genes, and 36 tRNA genes. Together with the four replicates of rRNA genes, the annotation shows five duplicated coding sequences (*ndhB*, *rpl2*, *rps7*, *rpl23*, and *ycf2)*, along with seven tRNA (*trnL-CAA*, *trnR-ACG*, *tnrI-GAU*, *trnV-GAC*, *tnrN-GUU*, *trnI-CAU*, and trnA-*UGC*). Trans-spliced *rps12* gene was annotated, as well as 10 cis-splicing genes including *clpP1*, *pafI*, *atpF*, *rpoC1*, *rps16*, *ndhA*, and both copies of *ndhB* and *rpl2* (Supplementary Figure 2).

**Figure 2.**
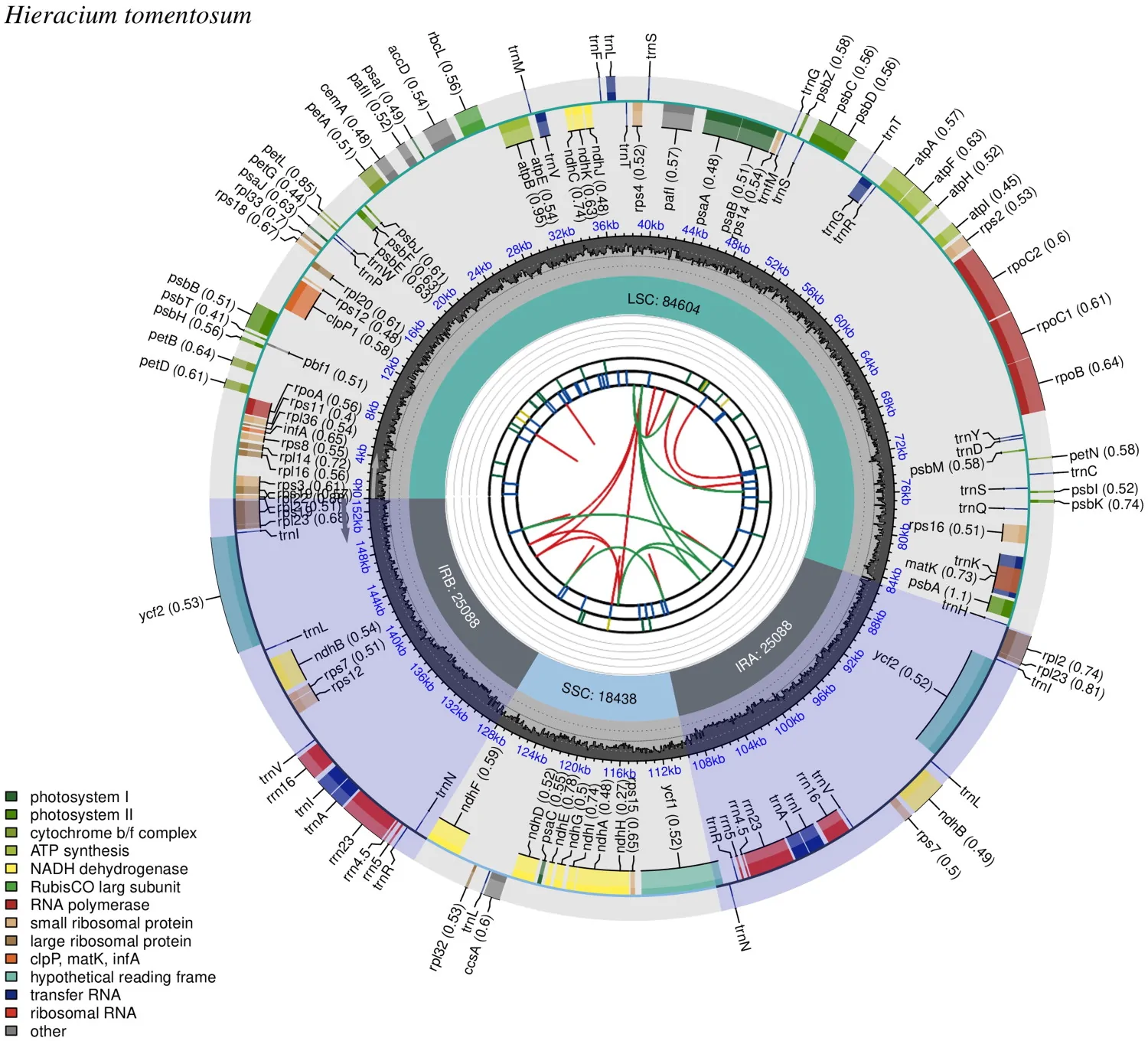
Chloroplast (cp) genome map of *Hieracium tomentosum*, highlighting all 127 annotated genes. The color of genes indicates their affiliation to the functional groups. The complete chloroplast genome shows the typical quadripartite structure with SSC, LSC, and two IR-B and -A. The circular genomic map of *H. tomentosum* with concentric rings highlighting different parameters. The outer ring presents annotated genes, color-coded indicating their functional groups. The second ring represents the organization of the chloroplast genome into the SS, LSC, and IR-B. The grey histogram depicts GC content, with the line graph depicts GC skew where green signifies positive, and red negative values, respectively. The innermost circle displays curved links connecting related genes, highlighting the genome’s structural organization. This representation was obtained with CPGView (Liu et al. 2023).

Maximum-likelihood phylogenomic tree placed diploid *H. tomentosum* within a well-supported genus *Hieracium* clade, which was recovered as closely related to genera *Schlagintweitia* and *Pilosella*, highlighting the monophyly of subtribe Hieraciinae (100/100 SH-alRT and bootstrap value). Hieraciinae was recovered as sister to the rest of Cichorieae subtribes, with Cichoriinae as the closest lineage, followed by a clade composed of Hypochaeridinae, Lactucinae, Hyoseridinae, and Crepidinae (Figure 3).

**Figure 3.**
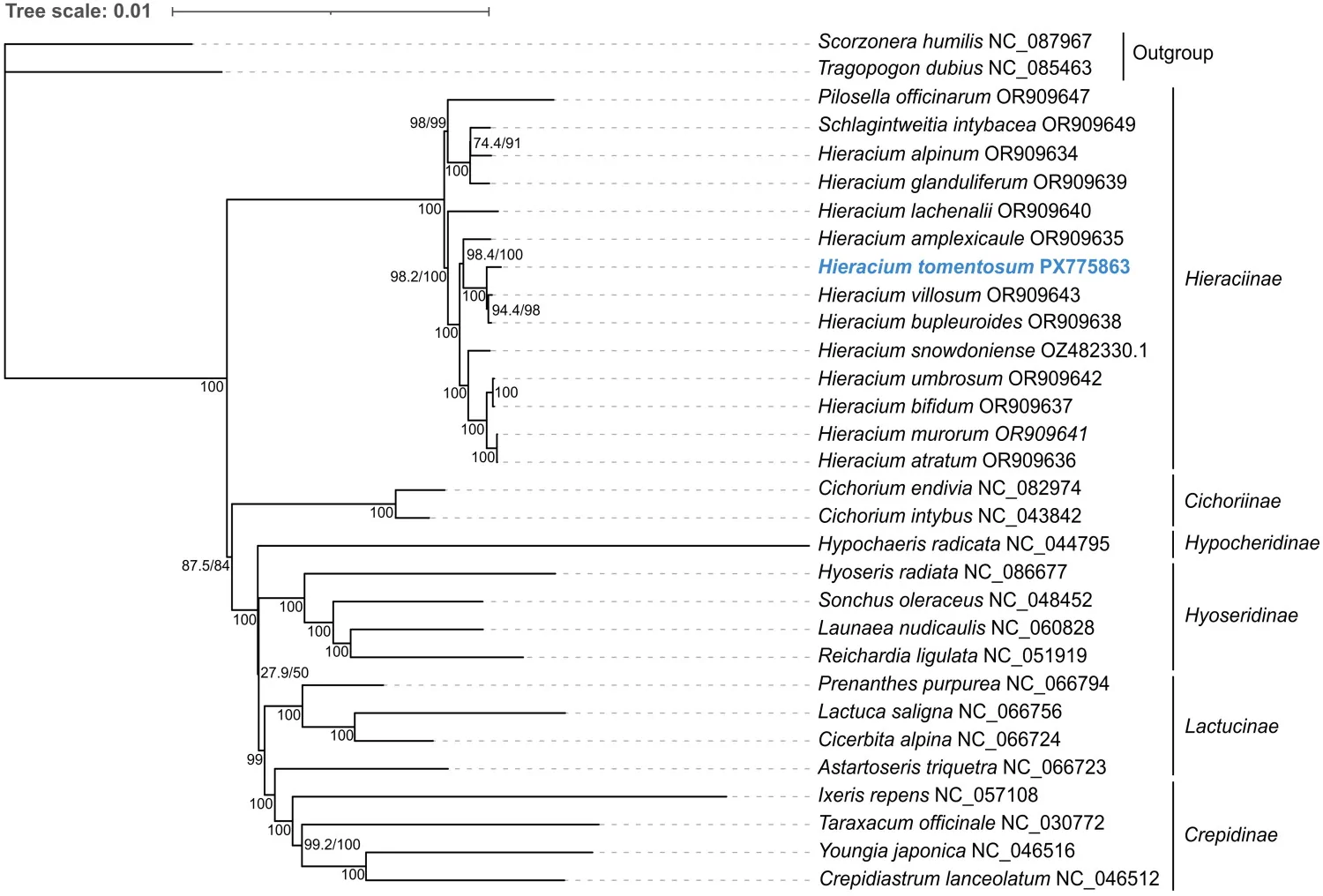
ML phylogenomic tree based on complete chloroplast genome of *Hieracium tomentosum* and 30 related representatives of tribe Cichorieae (Asteraceae) the SH-aLRT and bootstrap support values based on 1000 replicates are shown on each node (only showing one value if scores are the same). The tree is rooted at *Scorzonera humilis* NC_087967 (Xu et al. 2024) and *Tragopogon dubius* OR840963 (Huang et al. 2024). GenBank accessions for plastomes implemented in this study are: *Pilosella officinarum* OR909647, *Schlagintweitia intybacea* OR909649, *Hieracium alpinum* OR909634, *Hieracium glanduliferum* OR909639, *Hieracium lachenalii* OR909640, *Hieracium umbrosum* OR909642, *Hieracium bifidum* OR909637, *Hieracium murorum* OR909641, *Hieracium atratum* OR909636, *Hieracium amplexicaule* OR909635, *Hieracium villosum* OR909643, *Hieracium bupleuroides* OR909638 (all aforementioned accession are from Li et al. 2023, unpublished); *Hieracium snowdoniense* OZ482330; *Hieracium tomentosum* PX775863 (this study); *Cichorium intybus* NC_043842 (Yang et al. 2019), *Cichorium endivia* NC_082974 (Testone et al. 2023), *Hypochaeris radicata* NC_044795, *Hyoseris radiata* NC_086677, *Sonchus oleraceus* NC_048452 (Cho et al. 2019), *Launaea nudicaulis* NC_060828, *Reichardia ligulata* NC_051919 (Cho et al. 2020), *Prenanthes purpurea* NC_066794, *Lactuca saligna* NC_066756 (Chu et al. 2022), *Cicerbita alpina* NC_066724, *Astartoseris triquetra* NC_066723, *Ixeris repens* NC_057108 (Lee et al. 2021), *Taraxacum officinale* NC_030772 (Kim et al. 2016), *Youngia japonica* NC_046516 (Kim and Kim 2025), and *Crepidiastrum lanceolatum* NC_046512 (Kim et al. 2019). A phylogenetic tree illustrating evolutionary relationships among 31 species in the Cichorieae tribe (Asteraceae family), with each branch label referencing GenBank accession numbers, and a special color highlights the species presented in this study PX775863 *Hieracium tomentosum*. Values at the nodes are SH-aLRT support/ultrafast bootstrap support from IQTREE2, only depicting one value if the two scores are the same. The genetic scale at the top denotes distance of 0.01. Classification of Cichorieae subtribes is annotated at the right of tree tips.

## Discussion and conclusions

Phylogenomic analysis based on whole plastome sequences recovers *H. tomentosum* as clustering within Hieraciinae with strong overall support. Moreover, subtribe Hieraciinae is recovered as monophyletic, and sister to the rest of all subtribes in our phylogenomic tree. This is in apparent contrast to recent molecular phylogenies on Asteraceae (Kilian et al. 2009; Zhang et al. 2024) which depict the separation of Cichorieae into two large reciprocally monophyletic clades composed of Cichoriinae, Hieraciinae, and Microseridinae, and the rest (clade 4 and 5 in Kilian et al. 2009). The lack of plastome sequences encompassing this large diversity of species might account for the uncertain evolutionary relationships of Cichorieae subtribes, and their major divergence splits. Incomplete lineage sorting and introgression are common patterns occurring at sub-genus level in Cichorieae taxa (Kilian et al. 2009), and intergeneric hybridization within Hieraciinae has been also reported in cytoplasmic genome features (Krak et al. 2013; Chrtek et al. 2020). This scenario complicates even more the understanding of inter- and intra-genetic systematics of Cichorieae and the power of different methodologies in resolving phylogenies, which creates discrepancies between molecular and genomic, as well as nuclear and plastidial analysis (Fehrer et al. 2007; Krak et al. 2013; Wang et al. 2014; Zaika et al. 2020).

This study presents the complete chloroplast genome sequence of diploid *Hieracium tomentosum* as a representative of the megadiverse polyploid genus *Hieracium*. It provides a strong foundation for future research on Cichorieae and highlights the need for increased sampling and analyses to further clarify the evolutionary relationships of this intricate genus.

## Supplementary Material

SupplFigures_S1S2_HieraciumTomentosum_RV.pdf

## Data Availability

The genome sequence data that support the findings of this study are openly available in GenBank of NCBI at https://www.ncbi.nlm.nih.gov under the accession no. PX775863. The associated BioProject, SRA, and Bio-Sample numbers are PRJNA1373174, SRR36998144, and SAMN54689699, respectively.
