# Supplementary material for: The complete chloroplast genome of diploid Hieracium tomentosum (Asteraceae, Cichorieae)
Source: Mitochondrial DNA B Resour. 2026 Oct 22;11(11):1339–43. doi: 10.1080/23802359.2026.2728749 (PMC13700474; doi:10.1080/23802359.2026.2728749)
Supplement: Supplementary file 1 [file TMDN_A_2728749_SM1973.pdf]

Supplementary materials

Coverage depth plot of *Hieracium tomentosum* plastome

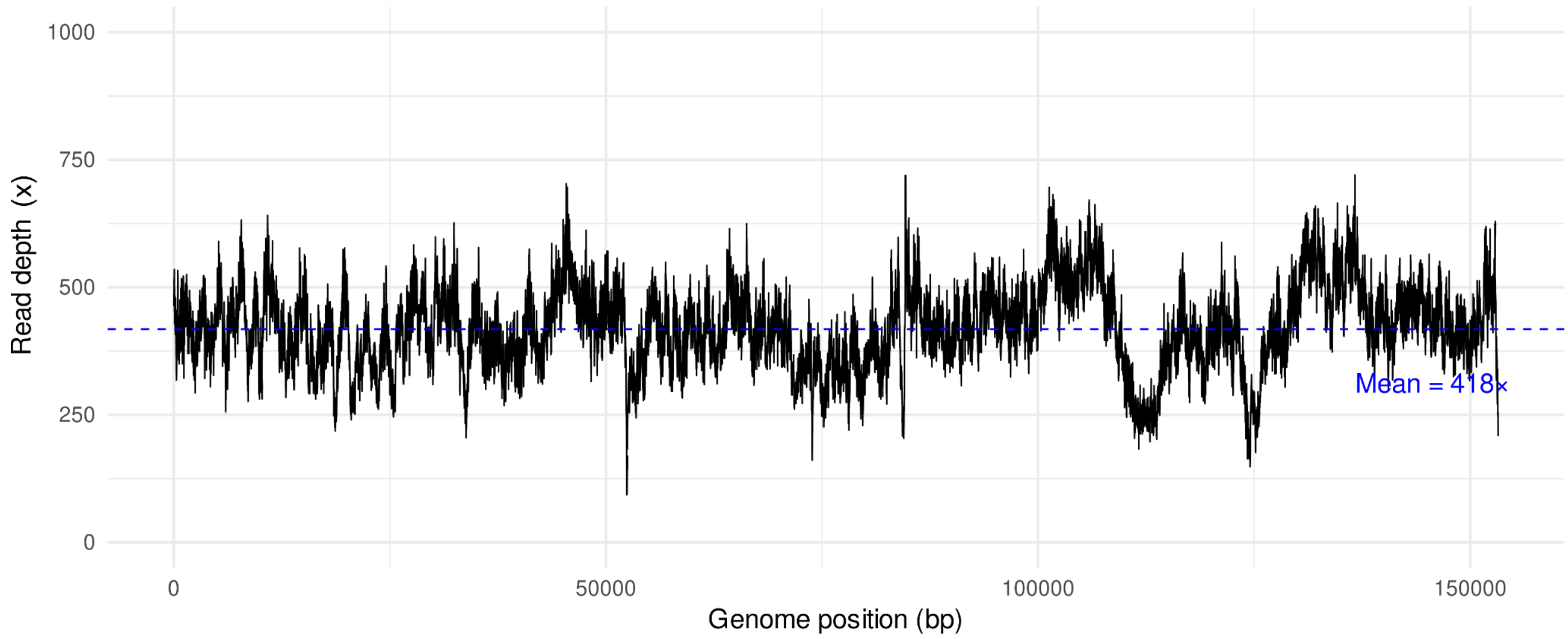

Figure S1. Coverage map of complete chloroplast genome of *Hieracium tomentosum*. Average sequencing read depth is 418x, highlighted in blue.

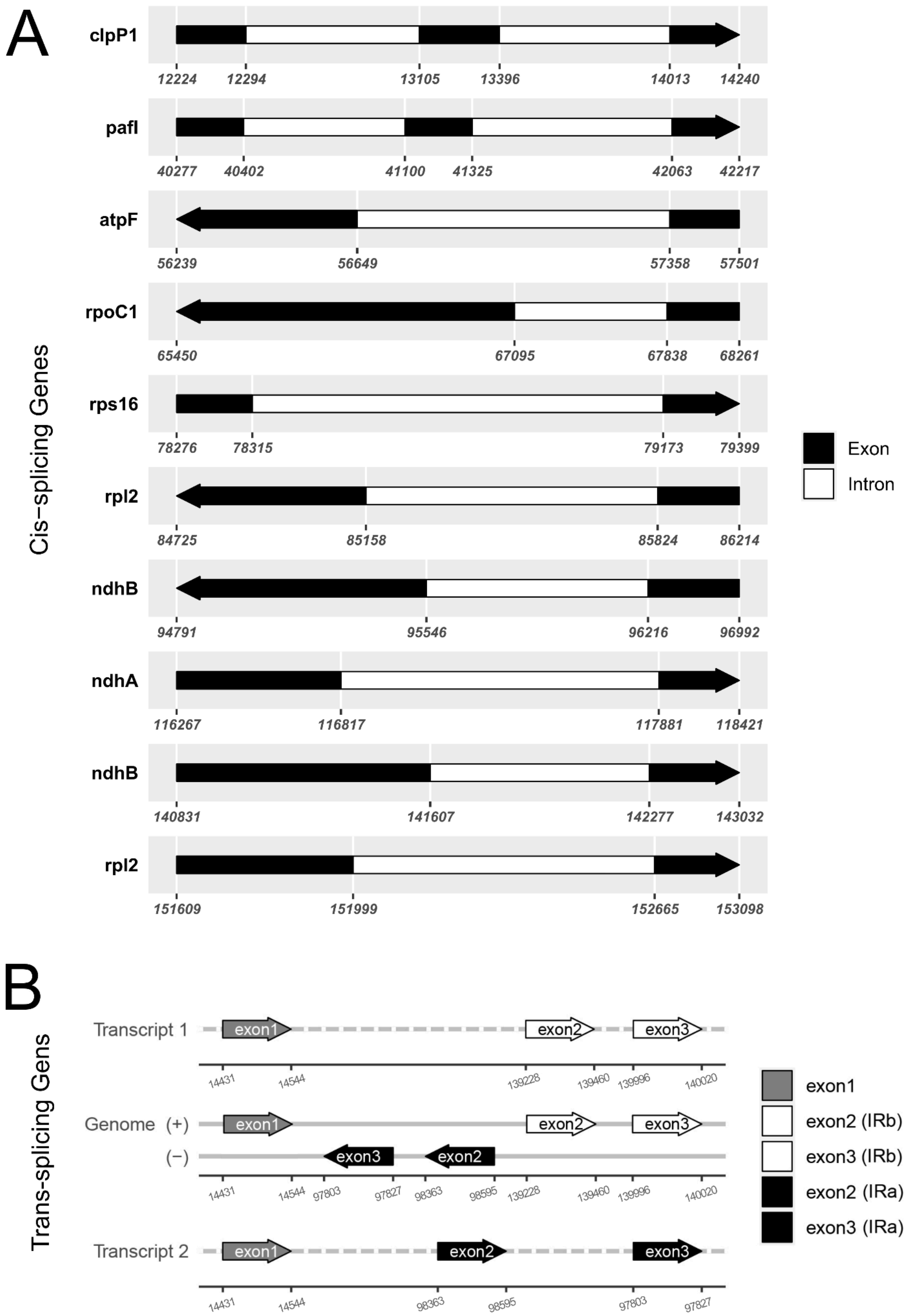

Figure S2. Schematic map of splicing genes of the chloroplast genome of *Hieracium tomentosum.* Arrows indicates the direction of transcription, and start and stop base positions are annotated at the horizontal axis. A: Structure of cis-splicing genes, with exons depicted in black and introns in white. B: Structure of trans-splicing gene *rps12.* This gene has 3 exons, two of which (exon2 and exon3) duplicated in each inverted repeat region (IR-B and IR-A).
